# Ototoxicity

**DOI:** 10.1289/ehp.113-a443

**Published:** 2005-07

**Authors:** Laurence D. Fechter, Benoit Pouyatos

**Affiliations:** Loma Linda VA Medical Center, Loma Linda, California, E-mail: Larry.fechter@med.va.gov

The January 2005 issue of *EHP* provided a much-needed overview of the prevalence of environment noise and its effects on health (Chepesiuk 2005; Manuel 2005; Schmidt 2005). Indeed, noise is pervasive and its adverse health effects are among the most common occupational injuries. Your consideration of noise-induced damage is especially welcome, given the strong focus of *EHP* on overexposure to chemical agents relative to overexposure to physical stimuli. Curiously absent from the discussion, however, was a review of the evidence that has accumulated over the past two decades concerning the ability of chemical agents to produce hearing impairment directly (ototoxicity) and to interact with noise exposure yielding either additive or synergistic impairment of the auditory apparatus. Research on such processes has received support in the United States from multiple agencies, including the National Institute of Environmental Health Sciences, the National Institute for Deafness and Other Communication Disorders, the National Institute for Occupational Safety and Health, and the U.S. Environmental Protection Agency.

Occupational epidemiology studies have demonstrated noise–chemical interactions in the workplace, and laboratory animal models have been effective in identifying ototoxicants, establishing dosimetry, identifying targets of toxicity, and determining the mechanisms for such ototoxicity. For example, occupational epidemiologic studies of Morata et al. (1997) demonstrated an excess risk of developing hearing loss among workers exposed to mixed solvents (mainly toluene) plus noise among printers compared with noise-exposed referent subjects or non-exposed matched controls. Similar studies have subsequently been published for styrene-exposed workers in the reinforced plastic industry (Morata et al. 2002; Sliwinska-Kowalska et al. 2003).

In laboratory animals, the pioneer experiments on the ototoxicity of solvents were initiated by Pryor and Rebert in the 1980s (e.g., Pryor et al. 1987; Rebert et al. 1983). Since these early studies, the ability of chemicals to directly disrupt auditory function has been established for trichloroethylene (Crofton et al. 1993; Fechter et al. 1998), toluene (Campo et al. 1999; Crofton et al. 1994; Johnson 1993), ethyl benzene (Cappaert et al. 2001), and styrene (Campo et al. 2001), among other agents. In addition, Lataye et al. (2001, 2003) have nicely identified the route by which solvents enter the cochlea and the pattern of damage that they produce in the inner ear.

Using developmental models, Rice and Gilbert (1992) demonstrated that methyl mercury exposure could impair auditory function in young primates. Also, hearing impairments have been reported for lead-exposed children (Osman et al. 1999; Schwartz and Otto 1987, 1991). Crofton and colleagues (Crofton et al. 1999, 2000; Lasky et al. 2002) demonstrated the ability of polychlorinated biphenyls to disrupt the development of the cochlea in rats by disrupting thyroid function.

In this laboratory, we have demonstrated that a series of chemical contaminants with potential to disrupt intrinsic antioxidant pathways or to enhance reactive oxygen species (ROS) generation can produce permanent hearing loss in the presence of noise. These agents include carbon monoxide (Fechter et al. 1987, 1988, 2000), hydrogen cyanide (Fechter et al. 2002), and acrylonitrile (Fechter et al. 2003; Pouyatos et al. 2005). This research provided evidence that intense noise can initiate ROS generation, resulting in cochlear damage. We hypothesized that even moderate noise levels, including noise close to permissible workplace exposure levels, may initiate ROS formation but that these are normally contained by antioxidant pathways. However, in the presence of pro-oxidant chemical agents, we demonstrated that even mild noise can yield oxidative stress leading to the death of sensory receptor cells for sound, the outer hair cells, and subsequent permanent impairment of auditory function (Fechter et al. 2000, 2002, 2003; Pouyatos et al. 2005). It is striking, although not surprising, that the auditory system is vulnerable to a range of chemical agents that initiate toxic processes that have been more fully studied in the brain and other organ systems.

The existing evidence has clear implications for both environmental and occupational health, and it highlights the continuing need for research on the issue. In Europe, the scientific information available has influenced public health policy. In February 2003, the European Parliament and the Council of the European Union (2003) published Directive 2003/10/EC on minimum safety requirements regarding the exposure of workers to noise. Ultimately, an increase in the awareness of the ototoxic potential of chemicals should improve preventive efforts and help reduce the risk of hearing loss.
